# Disruption of Fructose 1,6-Bisphosphatase 2 Proximity to MIC60 Correlates with Mitochondrial Ultrastructural Changes

**DOI:** 10.3390/cells15100942

**Published:** 2026-05-20

**Authors:** Łukasz Pietras, Marta Migocka-Patrzałek, Bartosz Budziak, Dariusz Rakus, Agnieszka Gizak

**Affiliations:** 1 Department of Molecular Physiology and Neurobiology, University of Wrocław, 50-335 Wrocław, Poland; lukasz.pietras2@uwr.edu.pl (Ł.P.); bartosz.budziak@uwr.edu.pl (B.B.); dariusz.rakus@uwr.edu.pl (D.R.); 2 Department of Animal Developmental Biology, University of Wroclaw, 50-335 Wrocław, Poland; marta.migocka-patrzalek@uwr.edu.pl

**Keywords:** FBP2, mitochondria, ultrastructure, MIC60, mitofilin, cardiomyocyte

## Abstract

**Highlights:**

**What are the main findings?**

- Tetrameric FBP2 coincides with mitochondrial ultrastructural defects.
- FBP2 tetramerization reduces its proximity to MIC60.

**What is the implication of the main finding?**

- FBP2 oligomer balance links metabolic cues to mitochondrial defects in disease.

**Abstract:**

Fructose 1,6-bisphosphatase 2 (FBP2) is a multifunctional protein whose cellular functions depend on its oligomeric state. Forced FBP2 tetramerization has been linked to microtubule disruption and impaired mitochondrial trafficking, accompanied by abnormal mitochondrial morphology. Here, we identify MIC60 (mitofilin), a core element of the mitochondrial contact site and cristae organizing system (MICOS), as a potential mediator of these effects. Using proximity ligation assay, protein crosslinking combined with mass spectrometry, and ultrastructural analysis, we demonstrate that FBP2 is in close proximity to MIC60 under basal conditions and this proximity is reduced upon FBP2 tetramerization or partial FBP2 depletion. Loss of this proximity coincides with marked remodeling of inner-membrane ultrastructure. These findings are consistent with a working model in which dimeric FBP2 contributes to the coordination of microtubule-dependent mitochondrial positioning with MICOS-linked intramitochondrial organization, providing a plausible mechanistic bridge between metabolic cues (AMP/NAD^+^) and mitochondrial structural integrity.

## 1. Introduction

**Figure 1:**
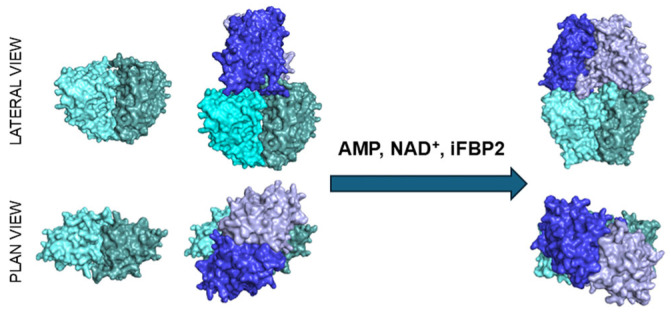
Oligomeric forms of FBP2 under different conditions. In the absence of physiological allosteric inhibitors (AMP and NAD^+^) or their functional mimetic iFBP2, FBP2 exists predominantly as dimers and R-state (active) tetramers (left side of the figure; lateral and plan views of the structures). The presence of these inhibitors shifts the equilibrium toward the T-state (inactive) tetramers (right side of the figure), unless FBP2 remains associated with protein partners. Molecular surfaces were generated using structures 5RT5 (dimer and R-state tetramer) and 5ET7 (T-state tetramer) deposited in the Protein Data Bank and visualized with PyMOL version 2.5.4. Individual FBP monomers are shown in different colors.

Fructose 1,6-bisphosphatase 2 (FBP2) is a key enzyme in glycogen de novo synthesis, classically known for its role in catalyzing the hydrolysis of fructose-1,6-bisphosphate to fructose-6-phosphate. However, in recent years, FBP2 has emerged as a multifunctional protein involved in non-metabolic cellular processes (e.g., [1,2,3,4,5,6]). Importantly, many of these functions appear to depend on the quaternary state of the protein. In cells, FBP2 can exist as a homotetramer in two conformational states: the active R-state and the inactive T-state, in which the upper dimer is rotated by approximately 13–20° relative to the lower one. Moreover, we have recently shown that FBP2 may also exist as a dimer. The dimeric form of FBP2 has the same kinetic parameters as its tetrameric forms, but it is insensitive to inhibition by allosteric effectors: AMP and NAD^+^. These distinct quaternary states expose different molecular surfaces, providing different docking interfaces for protein partners depending on the structural state of FBP2 (Figure 1) [7].

Consequently, our studies indicate that FBP2 may exist in distinct functional pools within the cell. In particular, a cytoplasmic pool of FBP2, likely involving R-state (active) tetramers interacting with aldolase A, appears to be linked to the regulation of glycolysis and glycogen synthesis from non-carbohydrate precursors. In contrast, tetrameric FBP2 tends to accumulate in the nucleus and is sensitive to allosteric inhibition, whereas a pool of FBP2 dimers associated with mitochondrial proteins exerts protective effects on the organelles under stress conditions. Such functional partitioning appears to be dynamically regulated by FBP2 binding partners, as well as metabolic and signaling cues [6,7,8].

Our previous work demonstrated that, in cardiomyocytes, dimeric FBP2 supports mitochondrial membrane potential and stabilizes the microtubule (MT) network. Conversely, chemically induced tetramerization of FBP2—using iFBP2, a synthetic FBP2 inhibitor that mimics the action of AMP/NAD^+^ by binding to the regulatory allosteric site [9]—or partial silencing of FBP2 leads to reduced mitochondrial motility, decreased mitochondrial membrane polarization, MT destabilization, and increased mitophagy. These effects were accompanied by a marked increase in tau phosphorylation at Thr231, a known marker of MT destabilization, and by enhanced interactions of FBP2 with the phosphorylated tau (pTau) and with MAP1B—both involved in cytoskeletal dynamics.

Given the gluconeogenic function of FBP2, the overabundance of inactive tetrameric FBP2 could, in principle, alter cellular energy balance. However, we found that providing excess glycolytic substrate did not modify the effect of FBP2 tetramerization on mitochondrial motility/network shape, indicating that these effects are independent of enzymatic activity and instead depend on FBP2 oligomeric state [6].

While these findings implicated FBP2 in the regulation of the MT–mitochondria interactions, they did not fully explain several striking observations, including the formation of donut-shaped mitochondria and mitochondrial aggregates upon FBP2 tetramerization or partial silencing [6]. These structures suggested deeper alterations in mitochondrial architecture, beyond those caused by tau/MAP1B modulation alone. Moreover, FBP2 has also been detected within the mitochondrial intermembrane space and matrix [4], raising the possibility that it may interact with intramitochondrial proteins that govern mitochondrial morphology and cristae structure.

To address this hypothesis, we performed a systematic search for proteins that are in functional proximity to both FBP2 and tubulin in control and iFBP2-treated HL-1 cardiomyocytes. This screen identified MIC60 (also known as mitofilin), a key component of the mitochondrial contact site and cristae organizing system (MICOS), as a novel putative FBP2-binding partner. MIC60 is essential for maintaining mitochondrial inner membrane structure [10] and, indirectly, for coordinating mitochondrial dynamics with cytoskeletal organization [11]. Here, we show that FBP2–MIC60 proximity is disrupted upon FBP2 tetramerization and that this correlates with marked alterations in mitochondrial ultrastructure, reminiscent of those seen in diseases associated with mitochondrial disorganization.

These findings expand the functional landscape of FBP2, positioning it as a molecular hub coordinating both cytoskeletal integrity and mitochondrial architecture and motility. Given that AMP and NAD^+^ levels increase in several pathophysiological conditions—including ischemia, heart failure, and neurodegeneration—understanding how FBP2 oligomerization affects MIC60 and the MT–mitochondria contacts may reveal new mechanistic insights into these disorders.

## 2. Materials and Methods

### 2.1. Cell Culture

The HL-1 cardiomyocyte cell line (a gift from Dr. W.C. Claycomb, Louisiana State University Health Science Center, New Orleans, LA, USA) was used in this study [12]. These immortalized, proliferative atrial cardiomyocytes were maintained in Claycomb medium enriched with 10% fetal bovine serum prescreened for HL-1 cells (cat. no TMS-016), 2 mM L-glutamine, 0.1 mM norepinephrine, and a standard antibiotic mix (penicillin/streptomycin). All reagents were obtained from Merck (Darmstadt, Germany). Culture dishes were coated with bovine fibronectin (5 μg/mL) and gelatin (0.02%) prior to seeding the cells. The cells were cultured at a constant temperature (37 °C), humidity (95%), and CO2 level (5%).

### 2.2. FBP2 Tetramerization and Partial Silencing

Chemical induction of FBP2 tetramerization was performed using 5-chloro-2-(N-(2,5-dichlorobenzenesulfonamido))-benzoxazole, abbreviated as iFBP2 (Cayman Chemical, Ann Arbor, MI, USA) at a final concentration of 5 µM for 1 h prior to subsequent analysis. Partial silencing of FBP2 expression was achieved via shRNA-mediated silencing using commercially available lentiviral particles (cat. no sc-45240-V; Santa Cruz Biotechnology, Dallas, TX, USA). The FBP2-silenced HL-1 cardiomyocytes used in this study are the same cell lines described in our earlier publication [6], in which silencing efficiency was verified and documented using FISH, immunofluorescence, and enzymatic activity assays. Accordingly, these validation data are not repeated here. Briefly, shRNA treatment resulted in partial FBP2 silencing, as indicated by ~40% reduction in FBP2 mRNA levels (FISH), ~36% decrease in FBP2 immunofluorescence signal, and a reduction of FBP2 enzymatic activity to approximately 40% of control levels.

### 2.3. Transmission Electron Microscopy (TEM)

For ultrastructural analysis of mitochondrial morphology, HL-1 cells were trypsinized, pelleted, and the pellet was fixed in 2.5% glutaraldehyde in 0.1 M phosphate buffer for 24 h, at 4 °C. The material was postfixed for 90 min in 1% osmium tetroxide, dehydrated through an ascending acetone series (30, 50, 70, 90, and 100% for 5 min each), and embedded in epoxy resin. All reagents were from Sigma-Aldrich, St. Louis, MO, USA.

Polymerization of the epoxy resin with embedded cells was performed at 45 °C for 24 h, and then 60 °C for 72 h. Samples were sectioned using a Leica Ultracut UCT (Leica, Wetzlar, Germany). Ultrathin sections (50 nm) were mounted on copper grids. Sections were contrasted with uranyl acetate and lead citrate before observation. Imaging was performed using a Talos L120C transmission electron microscope (Thermo Fisher Scientific, Waltham, MA, USA) at the Microscopy Techniques Laboratory of the Faculty of Biological Sciences.

### 2.4. Quantitative Analysis of Mitochondrial Shape and Ultrastructure Parameters in Electron Micrographs

Quantitative morphometric analysis of mitochondrial shape was performed on TEM images acquired at identical magnification. Image analysis was conducted using the open-source ImageJ/Fiji software version 1.54p [13], as follows: individual mitochondria were manually outlined with maximum precision using the freehand selection tool. For each traced mitochondrion, two parameters describing shape were extracted:

1. Aspect ratio (AR)—defined as the ratio of the major (longest) axis to the minor (shortest) axis of the fitted ellipse, reflecting elongation.
2. Circularity—calculated using the standard formula

$$circularity=(4\pi \times Area)/\;{Perimeter}^{2}$$

with values ranging from 0 (elongated or irregular) to 1.0 (perfect circle), indicating how closely the mitochondrial shape approximates a circle.

For quantitative analysis of mitochondrial ultrastructure, mitochondria displaying normal morphology and mitochondria with inner membrane abnormalities were counted independently in each analyzed image. The percentage of morphologically intact mitochondria per image was used for statistical analysis. In addition, the frequency of specific ultrastructural abnormalities (e.g., concentric or donut-shaped mitochondria, as described in [14,15]) was determined across all analyzed images. Since these abnormalities were not present in every image, the obtained distributions were not suitable for reliable statistical comparison.

At least *n* = 979 mitochondria were analyzed per condition, across 2 independent experiments.

### 2.5. Identification of Proteins Proximal to Both FBP2 and Tubulin via Crosslinking and Mass Spectrometry

To identify proteins proximal to both FBP2 and microtubules, in situ protein crosslinking was performed using NHS-SS-Diazirine (Succinimidyl 2-([4,4′-diaziridinyl]pentanamido)amino)dithiopropionate; SDAD; Thermo Fisher Scientific, Waltham, MA, USA). After crosslinking, using 365 nm UV light [4], a microtubule-enriched cellular fraction was isolated through established fractionation protocols [16]. The enriched fraction was resolved by SDS-PAGE, and three identical lanes were loaded in parallel. Two lanes were transferred to nitrocellulose membranes and probed with antibodies against FBP2 [6] and β-tubulin (cat. no ab15568; Abcam, Cambridge, UK), respectively. The third gel lane was silver-stained (according to [17]). Protein bands showing overlapping signals on both Western blots (i.e., containing both FBP2 and β-tubulin) were matched with corresponding bands on the silver-stained gel, excised, and subjected to in-gel tryptic digestion followed by mass-spectrometry-based protein identification. The analysis was performed commercially by the Mass Spectrometry Laboratory at the Institute of Biochemistry and Biophysics, Polish Academy of Sciences (Warsaw, Poland). Data processing and identification were conducted as described at https://mslab-ibb.pl/en/services/protein-identification (accessed 9 September 2025). The significance threshold for incorrect matches of a unique peptide to a protein sequence was set at the level of *p* < 0.003 (protein score 30 in Mascot Server Database).

### 2.6. Proximity Ligation Assay (PLA)

FBP2–MIC60, FBP2–dynein and TOMM20–dynein interactions under various conditions (control, iFBP2 treatment, partial FBP2 silencing) were assessed using the Duolink^®^ In Situ Proximity Ligation Assay (DuoLink^®^ In Situ Orange Starter Kit Mouse/Rabbit; Merck, Darmstadt, Germany), according to the manufacturer’s protocol. Fixed HL-1 cardiomyocytes were incubated with primary antibodies targeting FBP2 [6], MIC60 (cat. no MA5-47671; Invitrogen, Waltham, MA, USA), dynein (cat. no 14-9772-80; Invitrogen, Waltham, MA, USA) and TOMM20 (cat. no HPA011562; Merck, Darmstadt, Germany), followed by incubation with species-specific PLA probes. Signal amplification and detection steps were performed as per kit instructions. Images were acquired using the FV1000 confocal microscope (Olympus, Tokyo, Japan) with a 60× (oil, Plan SApo, NA = 1.35) objective and analyzed using the ImageJ/FIJI software version 1.54p [13]. The number of PLA signals (representing close proximity of the two proteins) was quantified per cell in at least 75 images per condition, across 3 independent experiments.

### 2.7. Delivery of Dimeric FBP2 Mutant Protein into HL-1 Cardiomyocytes

To assess the cellular effects of dimeric FBP2 independently of tetramer formation, we introduced a recombinant, dimeric mutant of human FBP2 into HL-1 cardiomyocytes. This variant carries a single amino acid substitution (L190G) that prevents its tetramerization by disrupting the interface required for higher-order oligomer assembly [7]. As a result, the L190G FBP2 mutant exists exclusively in a dimeric state and is resistant to allosteric inhibition by AMP, NAD^+^, or the commercial FBP2 inhibitor iFBP2. Recombinant L190G FBP2 protein was expressed in Escherichia coli and purified as previously described [7]. Protein delivery into mammalian cells was performed using the Chariot™ Protein Delivery Reagent (Active Motif Europe, Waterloo, Belgium), as we described in [7], with a slight modification: 0.5 µg of L190G FBP2 protein was included in the delivery mixture per well of a 24-well plate. Protein delivery success was determined by Western blot. Quantitative analysis revealed an approximately 2-fold increase in total FBP2 signal relative to control cells. The corresponding quantification graph and original membranes are presented in Supplementary Material S1a.

### 2.8. Statistical Analysis

Statistical analyses were performed using Statistica 13.3 software (StatSoft Polska/Tibco, Kraków, Poland) under the license of the University of Wrocław.

Normality was tested with the Shapiro–Wilk test (for sample sizes ≤ 30) and the Kolmogorov–Smirnov test (for sample sizes > 30). Equality of variances was then assessed using Levene’s test.

To determine the significance of differences between two groups with a normal distribution and homogeneous variances, Student’s *t*-test was applied. In cases of unequal variances, the Cochran–Cox correction was used with Student’s *t*-test. When at least one of the two groups did not follow a normal distribution, the Mann–Whitney U test was employed.

For comparisons involving three or more groups with a normal distribution and homogeneous variances, one-way ANOVA followed by the RIR-Tukey post hoc test was used. When variances were unequal, ANOVA with the F correction was applied. If at least one group did not follow a normal distribution, the Kruskal–Wallis ANOVA test was used instead, followed by Dunn’s multiple comparisons test. For cumulated distribution datasets, a Kolmogorov–Smirnov test for two populations was used. Differences were considered statistically significant at *p* ≤ 0.05.

The results are presented using three types of graphs: box-and-whisker plots, cumulative line graphs, and bar charts. The box-and-whisker plots depict quartiles, with the median marked by a line. Its value is shown on the graph when a non-parametric test (Mann–Whitney U or Kruskal–Wallis ANOVA) was used for statistical analysis. When a parametric test (Student’s *t*-test or ANOVA) was used, the mean is indicated by the symbol “x”. In bar charts, the height of each bar represents the mean value. Where possible, error bars representing one standard deviation are also included. The line graph only displays statistically significant differences in data distributions.

## 3. Results

### 3.1. FBP2 Silencing or Tetramerization Alters Mitochondrial Ultrastructure

In our previous paper, we showed that disturbance of FBP2–mitochondria interactions—either by partial silencing of FBP2 protein expression or chemical induction of tetramerization of this protein—disrupted mitochondrial dynamics and microtubule stability in HL-1 cardiomyocytes, which was accompanied by striking changes in mitochondrial network length and shape at the confocal microscopy level [6]. However, the underlying structural correlates of these changes remained unclear. To address this gap, we investigated whether changing the oligomeric state of FBP2 affects mitochondrial ultrastructure and geometry.

**Figure 2:**
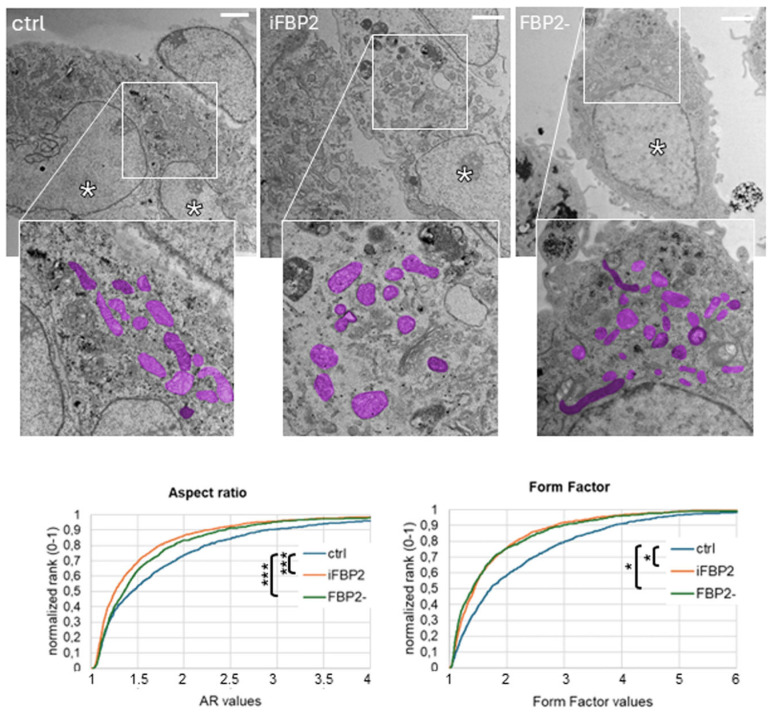
Mitochondrial shape in HL-1 cardiomyocytes. Representative TEM images showing overall mitochondrial morphology under control conditions (ctrl), after chemical tetramerization of FBP2 (iFBP2), and after partial FBP2 silencing (FBP2-). Cell profiles are shown, with nuclei marked by an asterisk (*). In the enlarged image insets, mitochondria are highlighted in magenta. Scale bar = 2 μm. Cumulative graphs present the quantification of mitochondrial shape descriptors, measured for each mitochondrion visible in the cell. At least 972 mitochondria were measured for each treatment. * *p* ≤ 0.5; *** *p* < 0.001.

Transmission electron microscopy revealed pronounced differences between mitochondria of control HL-1 cells and those depleted of dimeric FBP2 (either by iFBP2-induced tetramerization or shRNA-mediated partial silencing). Analysis of the shape of the individual mitochondria revealed a significant decrease in aspect ratio (AR, defined as the ratio of the major to minor axis of an object) and an increase in circularity, in cells treated with FBP2-tetramerizing factor (iFBP2 cells) or subjected to partial FBP2 silencing (FBP2- cells), compared to controls (Figure 2). These values reflected a shift toward shorter, more spherical mitochondria, consistent with mitochondrial fragmentation, confirming earlier observations from confocal microscopy [6], where mitochondrial shortening and swelling were observed under similar conditions.

**Figure 3:**
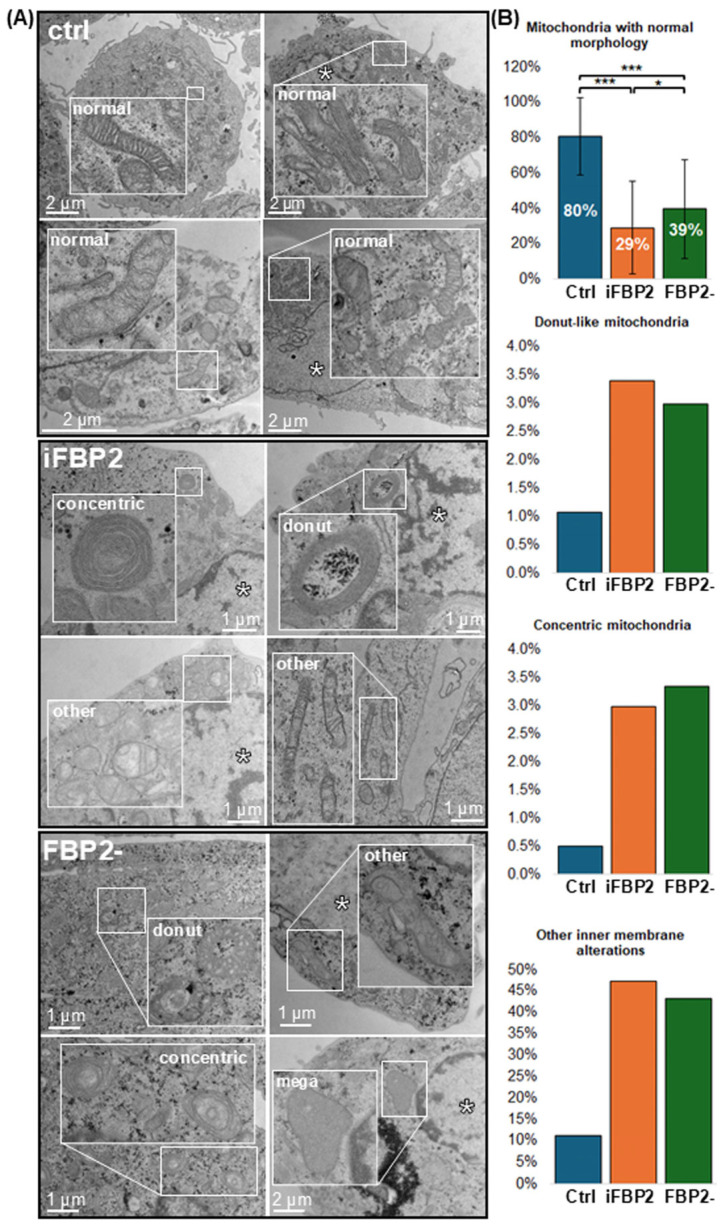
Mitochondrial inner structure in HL-1 cardiomyocytes. (**A**) Representative TEM images showing the inner membrane architecture of mitochondria under control conditions (ctrl), after chemical tetramerization of FBP2 (iFBP2), and after partial FBP2 silencing (FBP2-). An asterisk (*) indicates the nucleus, when visible. Mitochondria are labeled and classified according to their morphology as as normal, concentric, donut-shaped, megamitochondria (mega) or displaying other inner membrane alterations (other). (**B**) Quantification of mitochondrial inner membrane phenotypes. Graphs show the frequency of each mitochondrial type observed across all analyzed mitochondria (at least 979 for each condition). The graph labeled “Mitochondria with normal morphology” represents the proportion of morphologically intact mitochondria quantified independently for each image (at least 100 images for each condition) and was therefore used for statistical analysis. Data are presented as median ± standard deviation. * *p* < 0.05 *** *p* < 0.001. In contrast, the remaining categories represent the total frequency of specific ultrastructural abnormalities observed across all analyzed images, as these alterations were not present in every image analyzed.

In control conditions, the majority of mitochondria appeared elongated with well-organized cristae, although occasional signs of stress-related alterations were observed. However, the proportion of mitochondria with normal morphology was significantly higher in untreated than in either iFBP2-treated or FBP2-silenced cells, in which swollen, rounded mitochondria with irregular or fragmented cristae predominated. In these mitochondria, intercristal spaces were frequently widened, sometimes giving rise to vacuole-like structures. In FBP2-silenced cells, occasional megamitochondria were also observed, in line with prior reports [6]. In addition, concentric or “donut-shaped” mitochondrial profiles, which are commonly associated with inner membrane remodeling, were frequently observed in both experimental conditions. Figure 3 presents representative TEM images of abnormal mitochondrial structures, accompanied by quantitative analysis of their occurrence in each experimental condition.

Importantly, iFBP2 and FBP2- cells showed quite similar abnormalities in mitochondrial ultrastructure, suggesting a shared underlying factor: the absence or low level of FBP2 dimers.

Most of the observed changes could be linked to impaired mitochondrial membrane polarization and calcium buffering leading to an increased susceptibility to swelling—results of the lack of a protective pool of dimeric FBP2 [8,18,19]. However, not all abnormal mitochondria were swollen. Some retained elongated shapes but still showed disrupted internal architecture. This suggested that dimeric FBP2 may play a more direct role in shaping the inner mitochondrial membrane, beyond its role in stress buffering. The frequent occurrence of concentric mitochondrial profiles, a hallmark of inner membrane remodeling, strongly supported this hypothesis.

Together, these findings indicated that dimeric FBP2 may play a role in maintaining mitochondrial morphology and internal architecture, potentially by structural interactions within the mitochondrial membrane system.

### 3.2. Proteomics Identifies MIC60 as a Potential Mediator of FBP2-Driven Mitochondrial Remodeling

Given the pronounced mitochondrial alterations observed upon FBP2 tetramerization/partial silencing, we next sought to identify potential molecular mediators that might link FBP2 to the regulation of mitochondrial structure.

In a previous study, we performed a crosslinking-based proteomic screen aimed at identifying FBP2-associated mitochondrial proteins. The mitochondrial fraction isolated from crosslinker-treated HL-1 cells was separated by two “mirror” SDS-PAGEs (see also Materials and Methods section), and silver-stained protein bands corresponding to those detected with anti-FBP2 antibodies on a reference Western blot were analyzed by mass spectrometry [4]. Although numerous mitochondrial and some MT-related proteins were identified in that experiment, the purification procedure itself may have limited the detection of some FBP2-associated proteins, such as those localized specifically at the mitochondrial–cytoskeletal interface or transiently associated with contact sites.

**Figure 4:**
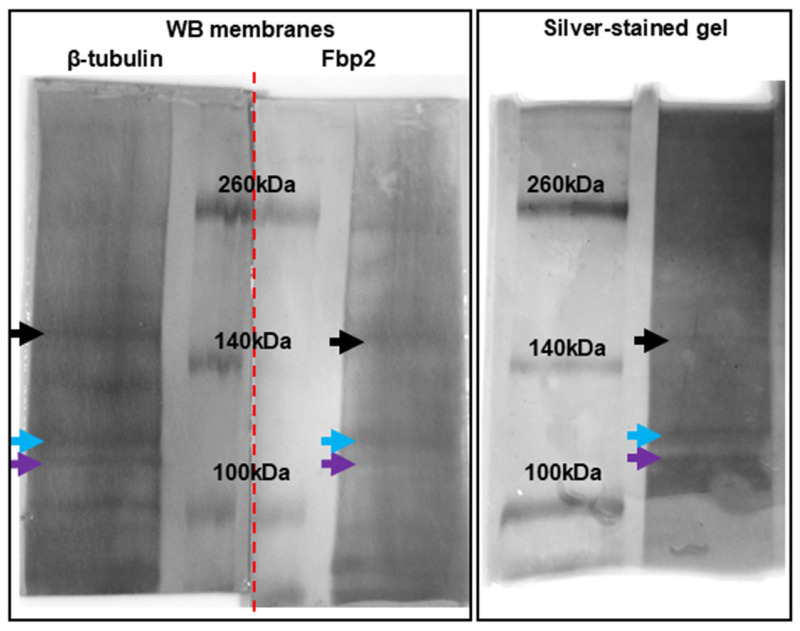
Dual Western blot screening of a microtubule-enriched protein fraction isolated from crosslinked HL-1 cardiomyocytes. (**Left panel**): Western blot membranes showing protein bands detected simultaneously with anti-FBP2 and anti-tubulin antibodies. This panel was assembled from two separate Western blot membranes placed side by side. The junction between the membranes is indicated by a red dashed line. (**Right panel**): silver-stained gel mirroring the one used for Western blot. Arrows indicate protein bands detected on both Western blot membranes. Each marked band was excised and subjected to mass spectrometry analysis. Corresponding bands on the membranes and the gel are marked with arrows of matching colors. Original pictures of the membranes and gel are presented as Supplementary Material S1b.

To address this, we repeated the crosslinking experiment using a microtubule-enriched fraction rather than isolated mitochondria and dual Western blot screening. The protein bands from silver-stained gel, corresponding to those detected simultaneously with anti-FBP2 or anti-tubulin antibodies on the blots, were subjected to mass spectrometry (Figure 4). In this experiment, we used untreated cells and cells pretreated with iFBP2. We did not use cells with partial silencing of FBP2 expression, as these contain both dimeric and tetrameric forms of FBP2—albeit at lower overall levels than control cells—so the same associations were expected, but with reduced intensity and thus harder to detect.

In the fraction isolated from control cells, three bands detected by both the antibodies were identified (Figure 4). In the fraction isolated from cells treated with FBP2 tetramerizing agent, despite several attempts, no such bands could be found), which is probably due to the fact that FBP2 tetramerization leads to a reduction in the association between FBP2 and tubulin [6].

This modified strategy enabled the identification of several proteins in proximity to both FBP2 and tubulin in control conditions. From the pool of identified proteins, we selected those implicated in microtubule-linked mitochondrial dynamics (fusion/fission, motility or mitophagy), based on literature data. These proteins are presented in Table 1. Some of them were already recognized as FBP2-interacting partners in previous studies [4,6]. All identified proteins can be found in Supplementary Material S2 and are also available under https://doi.org/10.34616/M61PGN, along with the list of unique peptides identified in each electrophoretic band.

Among these proteins we chose MIC60 (mitofilin) as a candidate protein simultaneously interacting with FBP2 and microtubules under control conditions. MIC60 is a key factor of the multisubunit MICOS complex, which is responsible for mitochondrial cristae dynamics and has multiple interaction partners in both the inner and outer mitochondrial membranes [35]. Silencing of the MIC60 gene is known to cause characteristic ultrastructural abnormalities of mitochondria, including concentric and donut-like profiles [36]. In our experiments, such concentric and donut-shaped mitochondria appeared 3-fold and 6-fold more frequently, respectively, in cells lacking a sufficient pool of FBP2 dimers compared to controls (Figure 2).

### 3.3. FBP2–MIC60 Proximity Is Disrupted by FBP2 Tetramerization and Partial Silencing

**Figure 5:**
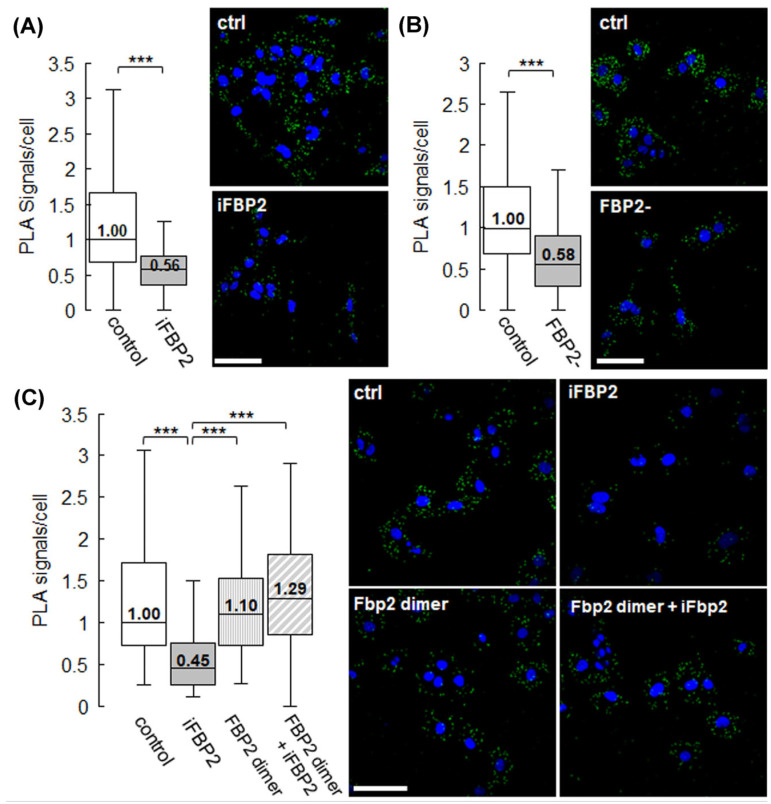
FBP2-MIC60 spatial proximity in different conditions. Per-cell quantification of FBP2-MIC60 PLA signals in HL-1 cells and representative confocal images, where green signal represents PLA-positive sites, nuclei are stained with DAPI (blue). (**A**) FBP2-MIC60 proximity in FBP2-tetramerizing-agent-treated cells (iFBP). (**B**) FBP2-MIC60 proximity in cells with partially silenced FBP2 expression (FBP2-). (**C**) FBP2-MIC60 proximity in cells transduced with non-tetramerizable FBP2 L190G mutein (FBP2 dimer), in control conditions and after treatment with the tetramerizing agent (iFBP). Data are presented as median and interquartile range. Scale bar = 40 μm. *** *p* < 0.001.

To validate the proximity between FBP2 and MIC60 in situ, we employed a Duolink proximity ligation assay (PLA) in HL-1 cardiomyocytes. In control conditions, a strong PLA signal was detected, indicating close proximity between the two proteins. However, both chemical tetramerization of FBP2 using iFBP2 and partial silencing of FBP2 expression led to a marked reduction in PLA signal (Figure 5), consistent with a decrease in their spatial association.

These findings support the proteomic results and reinforce the notion that dimeric, but not tetrameric, FBP2 is positioned in close proximity to MIC60. Loss of this spatial proximity correlates with the mitochondrial ultrastructural changes described above, suggesting a potential role for FBP2–MIC60 proximity in the maintenance of mitochondrial architecture and its spatial coordination with the cytoskeletal network.

To further demonstrate the involvement of the FBP2 dimer in this spatial association, we examined the FBP2–MIC60 proximity in cells transduced with a dimeric, non-tetramerizable FBP2 mutant, L190G [7]. The introduction of the mutant into the cells alone did not affect the number of interaction sites between FBP2 and MIC60. However, in its presence, tetramerization of the cellular FBP2 pool did not cause a decrease in the number of PLA signal sites (Figure 5).

In a separate experiment (Supplementary Material S3), the same mutant was able to rescue mitochondrial motility impaired by FBP2 tetramerization, suggesting that multiple mitochondrial functions depend on the dimeric form of FBP2.

### 3.4. FBP2 Tetramer Associates with Dynein and Disrupts Dynein–Mitochondria Coupling

**Figure 6:**
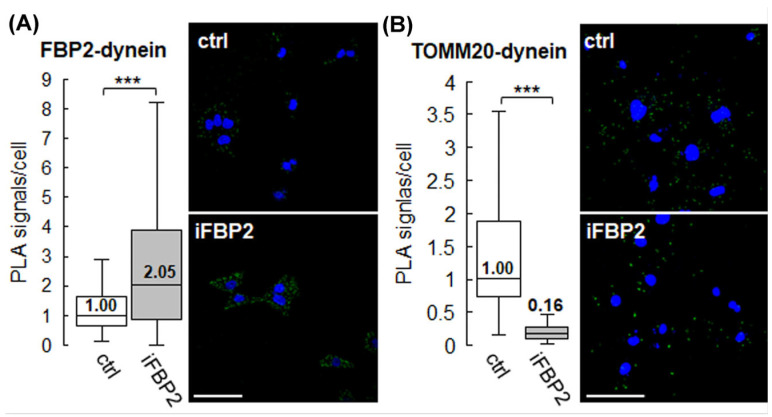
FBP2 tetramerization influences association with dynein and mitochondria. Per-cell quantification of PLA signal for (**A**) dynein-FBP2 and (**B**) dynein-TOMM20 together with representative confocal images, where green signal represents PLA-positive sites, and nuclei are stained in blue (DAPI). Ctrl—control conditions, iFBP2—FBP2-tetramerizing-agent-treated cells. Data are presented as median and interquartile range. Scale bar = 40 μm. *** *p* < 0.001.

The proteins identified by mass spectrometry included motor proteins responsible for the transport of mitochondria in both the anterograde and retrograde directions—kinesin and dynein, respectively. However, because a larger number of unique dynein peptides were identified, it was selected for further studies. Using the Duolink PLA technique, we obtained a confirmation of the close proximity of FBP2 and dynein in the cytoplasm of HL-1 cardiomyocytes. Chemically forced tetramerization of FBP2 resulted in a 2-fold increase in the number of PLA signals indicating protein proximity (Figure 6).

To assess dynein–mitochondria proximity we used antibodies against dynein and the mitochondrial marker TOMM20. Under physiological conditions, active retrograde transport should bring these proteins close enough to yield a positive signal. FBP2 tetramerization led to a >6-fold reduction in signal number (Figure 6), suggesting impaired dynein–mitochondria association. This disruption may contribute to decreased mitochondrial mobility.

Therefore, it appears that tetrameric FBP2 preferentially localizes in close proximity to dynein that has dissociated from mitochondria. This confirms and extends our previous findings on the effect of FBP2 tetramerization on its association with mitochondria/microtubule-related protein complexes [6].

## 4. Discussion

Our study provides new insight into the non-canonical functions of FBP2 in cardiomyocytes, further expanding role of its different oligomeric forms of beyond metabolic regulation.

Building on our earlier work on microtubule-dependent mitochondrial trafficking [6], we now show that mitochondrial ultrastructure changes reproducibly upon shifting the FBP2 dimer–tetramer balance or partially reducing FBP2 abundance.

Consistent results from PLA, crosslinking-MS, and ultrastructural analyses suggest that dimeric FBP2 resides in functional proximity to MIC60 (mitofilin) protein, and that disruption of this spatial arrangement correlates with cristae remodeling.

MIC60 is the key factor of the multisubunit MICOS complex that maintains the proper architecture of the inner mitochondrial membrane [35]. MIC60-deficient cells display markedly altered cristae structure, leading to enlarged mitochondria and impaired mitochondrial fusion and fission dynamics [37], which closely resemble the phenotypes observed upon forced FBP2 tetramerization ([6] and this paper). MIC60 interacts with several inner and outer membrane proteins, including SAM50, VDAC and ATP synthase [37,38,39,40], forming structural contact sites critical for mitochondrial architecture and function. All three of these proteins were also identified in FBP2-containing complexes (Supplementary Material S1 and [4]) suggesting that FBP2 may participate in—or influence—MICOS-associated intermembrane assemblies. Moreover, ATP synthase, known to interact with FBP2 in mitochondria, has been implicated in the spatial organization of MICOS [39,40].

In addition to its architectural role, MIC60 contributes to mitochondrial function and cell viability. Its deletion leads to lowered mitochondrial membrane potential, elevated ROS levels, and increased susceptibility to calcium-induced mitochondrial swelling and mPTP opening, ultimately resulting in higher rates of apoptosis [41,42]. Again, these are the same processes in which FBP2 is known to be involved [4,5,8], suggesting that FBP2–MIC60 proximity may be critical for the survival-regulating function of FBP2.

Interestingly, in Drosophila neurons, MIC60 depletion results in near-complete arrest of mitochondrial transport, indicating that MIC60 supports organelle motility by stabilizing outer membrane adaptor protein Miro that links mitochondria to motor complexes such as dynein and kinesin [11]. This mechanism aligns well with our findings in HL-1 cardiomyocytes, where depletion of FBP2 dimers associated with MIC60 leads to reduced mitochondrial motility and impaired dynein–mitochondria association ([6] and this paper).

Based on our findings, we propose a working model in which dimeric FBP2 participates in a transient protein assemblies linking microtubules and mitochondria and maintaining internal architecture of the organelles. In this framework, MIC60 emerges as a plausible structural hub in the vicinity of dimeric FBP2, alongside additional partners detected in our proximity and crosslinking data (e.g., VDAC, ATP synthase, SAM50 and microtubule-associated proteins).

Upon tetramerization—whether induced pharmacologically (iFBP2) or by physiological factors such as NAD^+^ or AMP—FBP2 loses its capacity to bind these protein partners. This loss coincides with disorganization of the mitochondrial network, disruption of cristae, depolarization of the organelles, and their adoption of rounded, concentric, or donut-like morphologies associated with impaired function and reduced mobility [6]. While our data do not demonstrate a direct causal role for MIC60, they support a MIC60-linked mechanism as a likely contributor to the ultrastructural phenotype.

Importantly, partial functional validation of this model was provided by an experiment involving a non-tetramerizable FBP2 mutant (L190G). Expression of this dimer-only variant prevented the disruption of FBP2–MIC60 proximity normally observed upon induction of FBP2 tetramerization.

Furthermore, it appears that, in control conditions, while dimeric FBP2 associates with mitochondria and microtubule-binding proteins, a cytosolic pool of tetrameric FBP2 may associate with dynein outside of the mitochondrial context. Upon forced tetramerization, however, FBP2 shows increased proximity to dynein accompanied by markedly reduced dynein–mitochondria proximity. One possible interpretation is that excess tetrameric FBP2 promotes retention of a fraction of dynein within the cytosol, thereby limiting its availability for mitochondrial transport-related assemblies. Since intracellular redistribution of dynein is a well-established mechanism regulating motor availability and cargo transport (reviewed in [43]), reduced dynein availability at the mitochondrial surface could contribute to impaired mitochondrial motility and abnormal mitochondrial morphology under conditions favoring FBP2 tetramerization. Nevertheless, this interpretation remains hypothetical and requires further mechanistic validation.

Consistently, the proteomic analysis of the crosslinked, MT-enriched cellular fraction suggests that the FBP2–dynein–microtubule association does not persist following forced FBP2 tetramerization, as no protein band was simultaneously detected by both anti-β-tubulin and anti-FBP2 antibodies.

Thus, the quaternary structure of FBP2 may serve as a regulatory switch coordinating cytoskeletal and mitochondrial integrity.

Our mechanistic findings gain further support from recent observations in human leukodystrophy linked to a pathogenic FBP2 variant (V115M). This mutation alters enzymatic activity and impairs non-canonical functions, including mitochondrial localization. Fibroblasts derived from affected patients exhibit disrupted mitochondrial networks, loss of FBP2–mitochondria colocalization, increased ROS levels, and decreased mitochondrial membrane potential [5]—all features reminiscent of the FBP2-silenced or tetramerized phenotype observed in our HL-1 model.

These data strongly suggest that mitochondrial stress and white matter injury observed in FBP2-related leukodystrophy may originate from failure of structural, non-enzymatic FBP2 functions, in particular its role in maintaining mitochondrial–cytoskeletal coupling. This is especially relevant in the context of demyelinating episodes during infancy, a period of intense oligodendrocyte activity and mitochondrial energy demand. Moreover, metabolic conditions known to stabilize tetrameric FBP2 (e.g., elevated NAD^+^ or AMP levels) occur in aging, ischemia, and neurodegenerative disorders—raising the possibility that disrupted FBP2 dimer:tetramer ratio may contribute more broadly to mitochondrial collapse and network fragmentation observed in cardiomyopathies, Alzheimer’s disease, or leukodystrophies of unclear etiology [44,45,46,47,48].

## 5. Conclusions

Taken together, our data indicate that FBP2 is part of a multiprotein network that connects mitochondria to the cytoskeleton. Our results from proximity ligation assays and protein crosslinking followed by mass spectrometry support the close spatial association of FBP2 with several proteins—e.g., tau, MAP1B, MIC60, dynein, tubulin as well as VDAC and ATP synthase (this paper and [4,6]). Importantly, FBP2 may not be associated with all these proteins simultaneously. Instead, it likely engages in distinct, context-dependent associations with selected partners—such as MIC60, MAP1B, VDAC or dynein—depending on cellular metabolic state (e.g., AMP or NAD^+^ levels). These transient assemblies may reflect specialized roles of FBP2 in coordinating cytoskeletal architecture, mitochondrial morphology, and organelle transport. However, these observations are primarily correlative and do not yet establish direct mechanistic relationships between FBP2 proximity patterns and the observed mitochondrial phenotypes.

Our findings raise the possibility that dysregulation of the FBP2 dimer–tetramer balance may contribute to disease contexts characterized by mitochondrial fragmentation and cristae defects, including cardiac and neurodegenerative conditions associated with altered AMP/NAD^+^ signaling. From this perspective, modulating the structural state of FBP2 may provide a testable approach to restore mitochondrial structure–transport coupling, although its therapeutic relevance and disease specificity will require dedicated in vivo validation.

**Figure 7:**
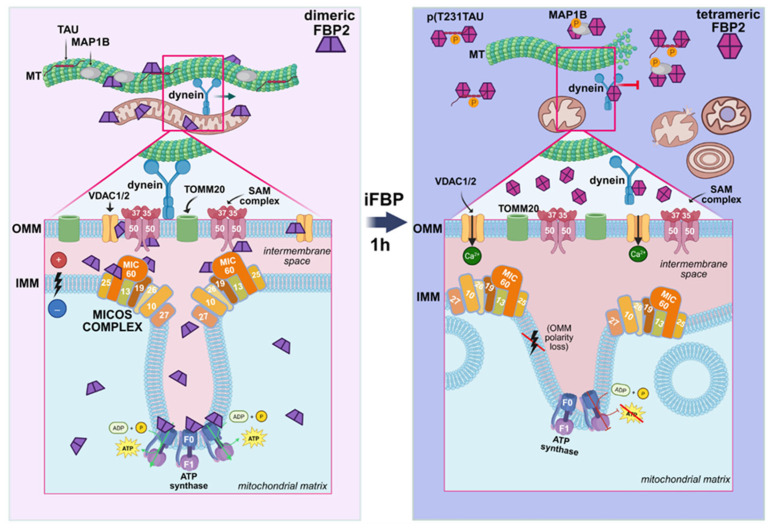
A summary of FBP2 proximity to mitochondrial and microtubule-interacting proteins under control conditions (**left panel**) and after forced tetramerization of the protein with iFBP2 (**right panel**). This figure is not intended to depict the precise composition or stoichiometry of FBP2-containing protein assemblies, which remain to be determined. Rather, it summarizes our current knowledge of FBP2’s association partners under the conditions studied. The functional relevance of these associations is discussed in detail in the main text.

A schematic summary of FBP2 associations with mitochondrial and microtubule-interacting proteins under control conditions and following forced tetramerization, integrating all experimental findings presented in this and our previous studies, is shown in Figure 7.

## 6. Study Limitations

This study has some limitations. Proximity ligation and crosslinking–mass spectrometry analyses indicate spatial proximity between FBP2 and MIC60 but do not demonstrate direct physical interaction or define the molecular interface. Rather, these approaches suggest that FBP2 localizes within functional proximity (≤40 nm) to these proteins, likely within larger structural or transport complexes.

Accordingly, future work involving structural mapping and mutagenesis will be required to determine whether FBP2 serves primarily as a regulator or stabilizing element within these complexes. In addition, although pharmacological and genetic manipulations were used to shift the FBP2 dimer–tetramer balance in cell cultures, the physiological regulation of FBP2 oligomerization and its impact on mitochondrial architecture under endogenous conditions remain to be fully characterized.

## Figures and Tables

**Table 1:** List of proteins in proximity to both FBP2 and β-tubulin, identified using mass spectrometry, that regulate selected mitochondrial functions—linked to MT-associated mitochondrial dynamics. Other known roles of these proteins are not included. Proteins that were also detected as FBP2-interacting partners in a previous study are marked with an asterisk. Proteins identified based on 1–2 unique peptides should be considered preliminary and interpreted with caution.

Protein	Uniprot Accession Number	Number of Identified Peptides	Mitochondrial-Dynamics-Related Function
ADP/ATP translocase 2 (SLC25A5) *	P51881	12	Involved in the correct initiation of mitophagy [20]
ATPase family AAA domain-containing protein 3 (ATAD3)	Q925I1	3	Participates in the regulation of the structure of mitochondrial cristae; silencing of its expression leads to the disruption of the structure of the cristae and reduced expression of the Mic60 protein [21]
CLIP-associated 2 (CLASP2)	Q8BRT1	1	Stabilizes microtubules and promotes their polymerization [22]
Dynein cytoplasmic 1 heavy chain 1 (DYNC1H1)	Q9JHU4	3	Responsible for the transport of mitochondria along microtubules [23]
Elongation factor 1-α-1 (EEF1A1)	P10126	12	Promotes microtubule stabilization [24]
Kinesin 1 heavy-chain (KIF5B)	Q61768	9	Responsible for the transport of mitochondria along microtubules [25]
MICOS complex Subunit mic60 (IMMT)	Q8CAQ8	20	A key component of the protein complex that organizes the shape of the inner mitochondrial membrane [26]
Microtubules associated protein 1b (MAP1B) *	P14873	1	Regulates the dynamics of microtubules, thereby influencing the distribution of mitochondria in the cell [27]
Prohibitin 1 (PHB1)	P67778	1	Takes part in the formation of the inner mitochondrial membrane and its compartmentalization [28]
Ras-related protein rab7a (RAB7A)	P51150	2	Participates in the formation of a phagophore around the mitochondria during mitophagy [29]
Solute carrier family 25 member 3 (SLC25A3)	Q8VEM8	6	Promotes the proteolysis of Opa1, thus participating in the inhibition of mitochondrial hyperfusion [30]
Sequestome-1 (SQSTM1)	Q64337	3	Regulator of mitophagy, likely in a Pink1/Parkin-independent pathway [31]
Sorting and assembly machinery component 50 homolog (SAMM50)	Q8BGH2	3	Interacts with the MICOS complex, participates in the formation of contact sites between the outer and inner mitochondrial membrane [32]
Voltage-dependent anion channel 1 (VDAC1)	Q60932	8	Participates in the initiation of mitochondrial swelling and apoptosis [33]
Voltage-dependent anion channel 2 (VDAC2) *	Q60930	8	Regulator of mitophagy, involved in the recruitment of Parkin to mitochondria by Pink1 [34]

## Data Availability

Proteomic data supporting the findings of this study are openly available in the RODBUK repository under the following DOI: https://doi.org/10.34616/M61PGN. All other data supporting the findings of this study are available from the corresponding author upon reasonable request.

## Supplementary Materials

The following supporting information can be downloaded at: https://www.mdpi.com/article/10.3390/cells15100942/s1, Supplementary Material S1: (a) Delivery of dimeric FBP2 mutant protein into HL-1 cardiomyocytes—original image of the WB membrane and quantification of the signal; (b) Original images of WB membranes and silver-stained gel (presented in Figure 4 of the main text); Supplementary Material S2: A complete list of proteins identified by mass spectrometry in Western blot bands detected simultaneously by anti-Fbp2 and anti-β-tubulin antibodies; Supplementary Material S3: Measurement of mitochondria velocity in HL-1 cardiomyocytes in the presence of dimer-only FBP2 mutant (L190G).

## Abbreviations

The following abbreviations are used in this manuscript:

AMP	Adenosine monophosphate
AR	Aspect ratio
FBP2	Fructose 1,6-bisphosphatase 2
FBP2-	Cells with partial silencing of FBP2 expression
FISH	Fluorescence in situ hybridization
iFBP	Cells treated with 5-chloro-2-(N-(2,5-dichlorobenzenesulfonamido))-benzoxazole
MAP1B	Microtubule-associated protein 1B
MIC60	Mitofilin
MICOS	Mitochondrial contact site and cristae organizing system
MS	Mass spectrometry
MT	Microtubule
NAD^+^	Nicotinamide adenine dinucleotide
PLA	Proximity ligation assay
ROS	Reactive oxygen species
SAM50	Sorting and Assembly Machinery 50
SDS-PAGE	Sodium dodecyl sulfate–polyacrylamide gel electrophoresis
TEM	Transmission electron microscopy
VDAC	Voltage-dependent anion channel

## References

[1] Duda P., Wójtowicz T., Janczara J., Krowarsch D., Czyrek A., Gizak A., Rakus D. (2020). Fructose 1,6-Bisphosphatase 2 Plays a Crucial Role in the Induction and Maintenance of Long-Term Potentiation. Cells.

[2] Duda P., Janczara J., McCubrey J.A., Gizak A., Rakus D. (2020). The Reverse Warburg Effect Is Associated with Fbp2-Dependent Hif1α Regulation in Cancer Cells Stimulated by Fibroblasts. Cells.

[3] Wang L., Wang J., Shen Y., Zheng Z., Sun J. (2022). Fructose-1,6-Bisphosphatase 2 Inhibits Oral Squamous Cell Carcinoma Tumorigenesis and Glucose Metabolism via Downregulation of c-Myc. Oxidative Med. Cell. Longev..

[4] Gizak A., Pirog M., Rakus D. (2012). Muscle FBPase Binds to Cardiomyocyte Mitochondria under Glycogen Synthase Kinase-3 Inhibition or Elevation of Cellular Ca^2+^ Level. FEBS Lett..

[5] Gizak A., Diegmann S., Dreha-Kulaczewski S., Wiśniewski J., Duda P., Ohlenbusch A., Huppke B., Henneke M., Höhne W., Altmüller J. (2021). A Novel Remitting Leukodystrophy Associated with a Variant in FBP2. Brain Commun..

[6] Pietras Ł., Stefanik E., Rakus D., Gizak A. (2022). FBP2-A New Player in Regulation of Motility of Mitochondria and Stability of Microtubules in Cardiomyocytes. Cells.

[7] Wiśniewski J., Piróg M., Hołubowicz R., Dobryszycki P., McCubrey J.A., Rakus D., Gizak A. (2017). Dimeric and Tetrameric Forms of Muscle Fructose-1,6-Bisphosphatase Play Different Roles in the Cell. Oncotarget.

[8] Hajka D., Budziak B., Rakus D., Gizak A. (2024). Neuronal Extracellular Vesicles Influence the Expression, Degradation and Oligomeric State of Fructose 1,6-Bisphosphatase 2 in Astrocytes Affecting Their Glycolytic Capacity. Sci. Rep..

[9] Von Geldern T.W., Lai C., Gum R.J., Daly M., Sun C., Fry E.H., Abad-Zapatero C. (2006). Benzoxazole Benzenesulfonamides Are Novel Allosteric Inhibitors of Fructose-1,6-Bisphosphatase with a Distinct Binding Mode. Bioorganic Med. Chem. Lett..

[10] Hessenberger M., Zerbes R.M., Rampelt H., Kunz S., Xavier A.H., Purfürst B., Lilie H., Pfanner N., van der Laan M., Daumke O. (2017). Regulated Membrane Remodeling by Mic60 Controls Formation of Mitochondrial Crista Junctions. Nat. Commun..

[11] Tsai P.-I., Papakyrikos A.M., Hsieh C.-H., Wang X. (2017). Drosophila MIC60/Mitofilin Conducts Dual Roles in Mitochondrial Motility and Crista Structure. Mol. Biol. Cell.

[12] Claycomb W.C., Lanson N.A., Stallworth B.S., Egeland D.B., Delcarpio J.B., Bahinski A., Izzo N.J. (1998). HL-1 Cells: A Cardiac Muscle Cell Line That Contracts and Retains Phenotypic Characteristics of the Adult Cardiomyocyte. Proc. Natl. Acad. Sci. USA.

[13] Schindelin J., Arganda-Carreras I., Frise E., Kaynig V., Longair M., Pietzsch T., Preibisch S., Rueden C., Saalfeld S., Schmid B. (2012). Fiji: An Open-Source Platform for Biological-Image Analysis. Nat. Methods.

[14] Vincent A.E., Ng Y.S., White K., Davey T., Mannella C., Falkous G., Feeney C., Schaefer A.M., McFarland R., Gorman G.S. (2016). The Spectrum of Mitochondrial Ultrastructural Defects in Mitochondrial Myopathy. Sci. Rep..

[15] Jenkins B.C., Neikirk K., Katti P., Claypool S.M., Kirabo A., McReynolds M.R., Hinton A. (2024). Mitochondria in Disease: Changes in Shapes and Dynamics. Trends Biochem. Sci..

[16] Lim A.C.B., Tiu S.-Y., Li Q., Qi R.Z. (2004). Direct Regulation of Microtubule Dynamics by Protein Kinase CK2. J. Biol. Chem..

[17] Yan J.X., Wait R., Berkelman T., Harry R.A., Westbrook J.A., Wheeler C.H., Dunn M.J. (2000). A Modified Silver Staining Protocol for Visualization of Proteins Compatible with Matrix-Assisted Laser Desorption/Ionization and Electrospray Ionization-Mass Spectrometry. Electrophoresis.

[18] Hajka D., Budziak B., Pietras Ł., Duda P., McCubrey J.A., Gizak A. (2021). GSK3 as a Regulator of Cytoskeleton Architecture: Consequences for Health and Disease. Cells.

[19] Pirog M., Gizak A., Rakus D. (2014). Changes in Quaternary Structure of Muscle Fructose-1,6-Bisphosphatase Regulate Affinity of the Enzyme to Mitochondria. Int. J. Biochem. Cell Biol..

[20] Hoshino A., Wang W.-J., Wada S., McDermott-Roe C., Evans C.S., Gosis B., Morley M.P., Rathi K.S., Li J., Li K. (2019). The ADP/ATP Translocase Drives Mitophagy Independent of Nucleotide Exchange. Nature.

[21] Peralta S., Goffart S., Williams S.L., Diaz F., Garcia S., Nissanka N., Area-Gomez E., Pohjoismäki J., Moraes C.T. (2018). ATAD3 Controls Mitochondrial Cristae Structure in Mouse Muscle, Influencing mtDNA Replication and Cholesterol Levels. J. Cell Sci..

[22] Mimori-Kiyosue Y., Grigoriev I., Lansbergen G., Sasaki H., Matsui C., Severin F., Galjart N., Grosveld F., Vorobjev I., Tsukita S. (2005). CLASP1 and CLASP2 Bind to EB1 and Regulate Microtubule Plus-End Dynamics at the Cell Cortex. J. Cell Biol..

[23] Pilling A.D., Horiuchi D., Lively C.M., Saxton W.M. (2006). Kinesin-1 and Dynein Are the Primary Motors for Fast Transport of Mitochondria in Drosophila Motor Axons. Mol. Biol. Cell.

[24] Moore R.C., Durso N.A., Cyr R.J. (1998). Elongation Factor-1α Stabilizes Microtubules in a Calcium/Calmodulin- Dependent Manner. Cell Motil. Cytoskelet..

[25] Wang C., Du W., Su Q.P., Zhu M., Feng P., Li Y., Zhou Y., Mi N., Zhu Y., Jiang D. (2015). Dynamic Tubulation of Mitochondria Drives Mitochondrial Network Formation. Cell Res..

[26] Mukherjee I., Ghosh M., Meinecke M. (2021). MICOS and the Mitochondrial Inner Membrane Morphology—When Things Get out of Shape. FEBS Lett..

[27] Bora G., Hensel N., Rademacher S., Koyunoǧlu D., Sunguroǧlu M., Aksu-Mengeş E., Balcl-Hayta B., Claus P., Erdem-Yurter H. (2020). Microtubule-Associated Protein 1B Dysregulates Microtubule Dynamics and Neuronal Mitochondrial Transport in Spinal Muscular Atrophy. Hum. Mol. Genet..

[28] Ban T., Kuroda K., Nishigori M., Yamashita K., Ohta K., Koshiba T. (2025). Prohibitin 1 Tethers Lipid Membranes and Regulates OPA1-Mediated Membrane Fusion. J. Biol. Chem..

[29] Kuchitsu Y., Fukuda M. (2018). Revisiting Rab7 Functions in Mammalian Autophagy: Rab7 Knockout Studies. Cells.

[30] Murata D., Roy S., Lutsenko S., Iijima M., Sesaki H. (2024). Slc25a3-Dependent Copper Transport Controls Flickering-Induced Opa1 Processing for Mitochondrial Safeguard. Dev. Cell.

[31] Yamada T., Dawson T.M., Yanagawa T., Iijima M., Sesaki H. (2019). SQSTM1/P62 Promotes Mitochondrial Ubiquitination Independently of PINK1 and PRKN/Parkin in Mitophagy. Autophagy.

[32] Chen L., Dong J., Liao S., Wang S., Wu Z., Zuo M., Liu B., Yan C., Chen Y., He H. (2022). Loss of Sam50 in Hepatocytes Induces Cardiolipin-Dependent Mitochondrial Membrane Remodeling to Trigger mtDNA Release and Liver Injury. Hepatology.

[33] Tomasello F., Messina A., Lartigue L., Schembri L., Medina C., Reina S., Thoraval D., Crouzet M., Ichas F., De Pinto V. (2009). Outer Membrane VDAC1 Controls Permeability Transition of the Inner Mitochondrial Membrane in Cellulo during Stress-Induced Apoptosis. Cell Res..

[34] Sun Y., Vashisht A.A., Tchieu J., Wohlschlegel J.A., Dreier L. (2012). Voltage-Dependent Anion Channels (VDACs) Recruit Parkin to Defective Mitochondria to Promote Mitochondrial Autophagy. J. Biol. Chem..

[35] Hu C., Shu L., Huang X., Yu J., Li L., Gong L., Yang M., Wu Z., Gao Z., Zhao Y. (2020). OPA1 and MICOS Regulate Mitochondrial Crista Dynamics and Formation. Cell Death Dis..

[36] Tsai P.I., Lin C.H., Hsieh C.H., Papakyrikos A.M., Kim M.J., Napolioni V., Schoor C., Couthouis J., Wu R.M., Wszolek Z.K. (2018). PINK1 Phosphorylates MIC60/Mitofilin to Control Structural Plasticity of Mitochondrial Crista Junctions. Mol. Cell.

[37] Cheng C., Chen M., Sun J., Xu J., Deng S., Xia J., Han Y., Zhang X., Wang J., Lei L. (2024). The MICOS Complex Subunit Mic60 Is Hijacked by Intracellular Bacteria to Manipulate Mitochondrial Dynamics and Promote Bacterial Pathogenicity. Adv. Sci..

[38] Tang J., Zhang K., Dong J., Yan C., Hu C., Ji H., Chen L., Chen S., Zhao H., Song Z. (2020). Sam50-Mic19-Mic60 Axis Determines Mitochondrial Cristae Architecture by Mediating Mitochondrial Outer and Inner Membrane Contact. Cell Death Differ..

[39] Stephan T., Brüser C., Deckers M., Steyer A.M., Balzarotti F., Barbot M., Behr T.S., Heim G., Hübner W., Ilgen P. (2020). MICOS Assembly Controls Mitochondrial Inner Membrane Remodeling and Crista Junction Redistribution to Mediate Cristae Formation. EMBO J..

[40] Eydt K., Davies K.M., Behrendt C., Wittig I., Reichert A.S. (2017). Cristae Architecture Is Determined by an Interplay of the MICOS Complex and the F1FO ATP Synthase via Mic27 and Mic10. Microb. Cell.

[41] Madungwe N.B., Feng Y., Lie M., Tombo N., Liu L., Kaya F., Bopassa J.C. (2018). Mitochondrial Inner Membrane Protein (Mitofilin) Knockdown Induces Cell Death by Apoptosis via an AIF-PARP-Dependent Mechanism and Cell Cycle Arrest. Am. J. Physiol.-Cell Physiol..

[42] Tombo N., Imam Aliagan A.D., Feng Y., Singh H., Bopassa J.C. (2020). Cardiac Ischemia/Reperfusion Stress Reduces Inner Mitochondrial Membrane Protein (Mitofilin) Levels during Early Reperfusion. Free Radic. Biol. Med..

[43] Cianfrocco M.A., DeSantis M.E., Leschziner A.E., Reck-Peterson S.L. (2015). Mechanism and Regulation of Cytoplasmic Dynein. Annu. Rev. Cell Dev. Biol..

[44] Baloyannis S.J. (2011). Mitochondria Are Related to Synaptic Pathology in Alzheimer’s Disease. Int. J. Alzheimer’s Dis..

[45] Roosendaal S.D., van de Brug T., Alves C.a.P.F., Blaser S., Vanderver A., Wolf N.I., van der Knaap M.S. (2021). Imaging Patterns Characterizing Mitochondrial Leukodystrophies. AJNR Am. J. Neuroradiol..

[46] Ahuja P., Wanagat J., Wang Z., Wang Y., Liem D.A., Ping P., Antoshechkin I.A., Margulies K.B., Maclellan W.R. (2013). Divergent Mitochondrial Biogenesis Responses in Human Cardiomyopathy. Circulation.

[47] Galloway C.A., Yoon Y. (2015). Mitochondrial Dynamics in Diabetic Cardiomyopathy. Antioxid. Redox Signal..

[48] Kanzaki Y., Terasaki F., Okabe M., Otsuka K., Katashima T., Fujita S., Ito T., Kitaura Y. (2010). Giant Mitochondria in the Myocardium of a Patient with Mitochondrial Cardiomyopathy: Transmission and 3-Dimensional Scanning Electron Microscopy. Circulation.

