# Supplementary material for: Disruption of Fructose 1,6-Bisphosphatase 2 Proximity to MIC60 Correlates with Mitochondrial Ultrastructural Changes
Source: Cells. 2026 May 20;15(10):942. doi: 10.3390/cells15100942 (PMC13204092; doi:10.3390/cells15100942)
Supplement: Supplementary file 1 [file cells-15-00942-s001.zip › Supplementary Material 3.pdf]

Supplementary Materials 3
Measurement of mitochondria velocity in HL-1 cardiomyocytes in the presence of with dimer-only FBP2 mutant (L190G)

The HL-1 cardiomyocytes were seeded on 35 mm glass-bottom dish (cat. no 801002, Nest, Jiangsu, China), 72h before the imaging. Mitochondria were visualized using MitoTracker™ Deep Red FM (cat. no M22426, Invitrogen, Carlsbad, CA, USA). The cells were incubated for 20 minutes with MitoTracker™ diluted to 100 nM in culture medium, then washed twice, incubated in fresh culture medium and imaged using TimeScan option with the Olympus FV1000 confocal microscope (Olympus, Tokyo, Japan). 60s-lasting time-lapse videos were created with 3x-digital zoom and analyzed using FV-10-ASW 4.2 Viewer software (Olympus, Tokyo, Japan). To avoid measurement of random movement of mitochondria caused by cytoplasm flow, mitochondria that have moved at least 2 µm were analyzed. In each condition, the speed of at least 784 mitochondria was quantified across 3 independent experiments.

Previously, it was demonstrated that both chemically forced tetramerization and partial silencing of FBP2 result in a significant reduction in mitochondrial transport velocity in HL-1 cardiomyocytes [1]. This effect was hypothesized to stem from the fact that dimeric FBP2 localizes to mitochondria [2]; thus, reduction of its cellular pool – through either silencing or forced tetramerization – leads to decreased FBP2–mitochondria colocalization.
To test this hypothesis directly, we performed a rescue experiment using a non-tetramerizable FBP2 mutant (L190G). HL-1 cells were transduced with the recombinant L190G FBP2 protein and treated with an FBP2-tetramerizing agent – iFBP (as described in the Materials and Methods of the main text). Parallel controls included untreated cells and cells treated with iFBP2 alone (without L190G-FBP2 delivery). In agreement with our previous findings [1], iFBP2-treated cells showed approximately 25% reduction in average mitochondrial velocity (Supplementary fig. 1).

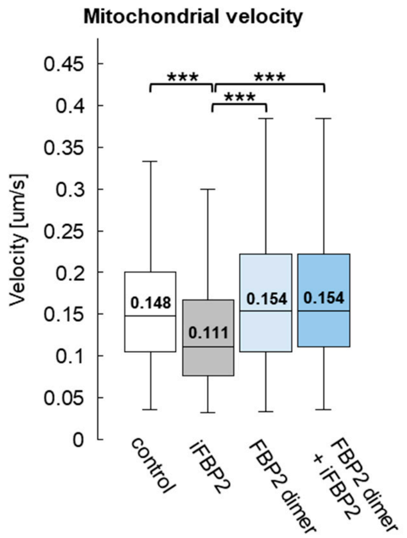

Supplementary Figure S1. **Quantification of mitochondrial velocity in HL-1 cardiomyocytes**
Control – mitochondrial velocity in HL-1 cells after application of Chariot™ Protein Delivery Reagent, without added protein; iFBP2 – control cells treated with 5 µM FBP2 tetramerizing agent; FBP2 dimer – cells transduced with L190G FBP2 (the non-tetramerizable FBP2 mutein) using Chariot™ Protein Delivery Reagent; FBP2 dimer + iFBP2 – transduced cells treated with 5 µM FBP2 tetramerizing agent. Data are presented as median and interquartile range. ***p< 0.001.

Notably, delivery of L190G-FBP2 alone did not affect mitochondrial velocity compared to untreated controls. However, in transduced cells treated with iFBP2, mitochondrial velocity remained comparable to untreated controls and was significantly higher than in iFBP2-treated cells without L190G rescue. This result indicates that the presence of dimeric FBP2 is sufficient to prevent the transport-impairing effects of endogenous FBP2 tetramerization (Supplementary fig. 1).
Moreover, it suggests that the previously observed mitochondrial defects following FBP2 tetramerization or partial silencing result from the insufficient amount of the dimeric form, rather than the presence of the tetramer per se.

1. Pietras, Ł.; Stefanik, E.; Rakus, D.; Gizak, A. FBP2-A New Player in Regulation of Motility of Mitochondria and Stability of Microtubules in Cardiomyocytes. *Cells* **2022**, *11*, 1710, doi:10.3390/cells11101710.
2. Gizak, A.; Pirog, M.; Rakus, D. Muscle FBPase Binds to Cardiomyocyte Mitochondria under Glycogen Synthase Kinase-3 Inhibition or Elevation of Cellular Ca 2+ Level. *FEBS Letters* **2012**, *586*, 13–19, doi:10.1016/j.febslet.2011.11.032.
