# Supplementary material for: Disruption of Fructose 1,6-Bisphosphatase 2 Proximity to MIC60 Correlates with Mitochondrial Ultrastructural Changes
Source: Cells. 2026 May 20;15(10):942. doi: 10.3390/cells15100942 (PMC13204092; doi:10.3390/cells15100942)
Supplement: Supplementary file 1 [file cells-15-00942-s001.zip › Supplementary Material 2.pdf]

Supplementary Material 2.

A complete list of proteins identified by mass spectrometry in Western blot bands detected simultaneously by anti-Fbp2 and anti-β-tubulin antibodies

The significance threshold for incorrect matches of a unique peptide to a protein sequence was set at the level of $p<0.003$ (protein score 30 in Mascot Server Database). Proteins identified based on fewer than four peptides were excluded from this list to avoid unnecessary expansion of the table. Proteins previously detected in proteomic screen of FBP2-interacting mitochondrial proteins in HL-1 cells [1] are marked by asterisk (*).

110 kDa band

Protein	Gene	Molecular weight (kDa)	UniProt accession number	Number of identified peptides
ADP/ATP translocase 2*	Slc25a5	33138	P51881	12
ADP/ATP translocase 1*	Slc25a4	33111	P48962	11
Voltage-dependent anion-selective channel protein 2*	Vdac2	32340	Q60930	8
Voltage-dependent anion-selective channel protein 1	Vdac1	32502	Q60932	8
Glyceraldehyde-3-phosphate dehydrogenase	Gapdh	36072	P16858	4
Heterogeneous nuclear ribonucleoprotein A1	Hnrnpa1	34289	P49312	4
ATP synthase subunit gamma, mitochondrial	Atp5f1c	32979	Q91VR2	6
Voltage-dependent anion-selective channel protein 3	Vdac3	31076	Q60931	5
Cytochrome c1, heme protein, mitochondrial	Cyc1	35533	Q9D0M3	5
Heterogeneous nuclear ribonucleoproteins A2/B1	Hnrnpa2b1	37437	O88569	6
60S ribosomal protein L6	Rpl6	33546	P47911	6
Histone H1.4	H1-4	21964	P43274	4
Sodium/potassium-transporting ATPase subunit beta-1	Atp1b1	35571	P14094	5
Histone H1.2	H1-2	21254	P15864	4
Annexin A2	Anxa2	38937	P07356	6

120 kDa band

Protein	Gene	Molecular weight (kDa)	UniProt accession number	Number of identified peptides
ADP/ATP translocase 2*	Slc25a5	33138	P51881	9
Actin	Actb	42052	P60710	19
Vimentin	Vim	53712	P20152	26
Actin, alpha cardiac muscle 1	Actc1	42334	P68033	17
Actin, aortic smooth muscle	Acta2	42381	P62737	17
Elongation factor 1-alpha 1	Eef1a1	50424	P10126	12
Glyceraldehyde-3-phosphate dehydrogenase	Gapdh	36072	P16858	10

Peripherin	Prph	54349	P15331	14
cAMP-dependent protein kinase type I-alpha regulatory subunit	Prkar1a	43443	Q9DBC7	10
Sorting and assembly machinery component 50 homolog	Samm50	52230	Q8BGH2	10
Alpha-enolase	Eno1	47453	P17182	8
Dihydrolipoyllysine-residue succinyltransferase component of 2-oxoglutarate dehydrogenase complex, mitochondrial	Dlst	49306	Q9D2G2	6
Voltage-dependent anion-selective channel protein 2*	Vdac2	32340	Q60930	9
Serpin H1	Serpinh1	46618	P19324	8
Elongation factor 1-alpha 2	Eef1a2	50764	P62631	7
Voltage-dependent anion-selective channel protein 1	Vdac1	32502	Q60932	7
60S ribosomal protein L4	Rpl4	47409	Q9D8E6	10
Keratin, type I cytoskeletal 17	Krt17	48417	Q9QWL7	15
Lysosome-associated membrane glycoprotein 1	Lamp1	44293	P11438	6
ADP/ATP translocase 1*	Slc25a4	33111	P48962	9
Heterogeneous nuclear ribonucleoproteins A2/B1	Hnrnpa2b1	37437	O88569	7
Heterogeneous nuclear ribonucleoprotein A1	Hnrnpa1	34289	P49312	5
Cytochrome c1, heme protein, mitochondrial	Cyc1	35533	Q9D0M3	6
Blood vessel epicardial substance	Bves	41502	Q9ES83	6
Cytochrome b-c1 complex subunit 2, mitochondrial	Uqcrc2	48262	Q9DB77	5
Voltage-dependent anion-selective channel protein 3	Vdac3	31076	Q60931	5
Heterogeneous nuclear ribonucleoprotein A3	Hnrnpa3	39856	Q8BG05	4
Kinesin-1 heavy chain	Kif5b	110225	Q61768	9
Popeye domain-containing protein 2	Popdc2	41966	Q9ES82	5
Inhibitor of nuclear factor kappa-B kinase-interacting protein	Ikbip	42563	Q9DBZ1	4
Histone H1.4	H1-4	21964	P43274	5
60S ribosomal protein L15	Rpl15	24245	Q9CZM2	5
Calreticulin	Calr	48136	P14211	6
ATP synthase subunit gamma, mitochondrial	Atp5f1c	32979	Q91VR2	5
Phosphate carrier protein, mitochondrial	Slc25a3	40063	Q8VEM8	6
Histone H1.2	H1-2	21254	P15864	4
LIM zinc-binding domain-containing Nebulette	Nebl	31492	Q9DC07	4
Lysosome-associated membrane	Lamp2	46165	P17047	6

glycoprotein 2				
Calsequestrin-2	Casq2	48203	O09161	4
Lysocardiolipin acyltransferase 1	Lclat1	44827	Q3UN02	4
Very-long-chain (3R)-3-hydroxyacyl-CoA dehydratase 3	Hacd3	43274	Q8K2C9	4
60S ribosomal protein L13	Rpl13	24348	P47963	4
60S ribosomal protein L6	Rpl6	33546	P47911	5
Elongation factor 1-gamma	Eef1g	50371	Q9D8N0	4
Peroxiredoxin-1	Prdx1	22390	P35700	5
60S ribosomal protein L18	Rpl18	21688	P35980	4
Surfeit locus protein 4	Surf4	30589	Q64310	4
60S acidic ribosomal protein P0	Rplp0	34366	P14869	4
Dolichyl-diphosphooligosaccharide--protein glycosyltransferase 48 kDa subunit	Ddost	49225	O54734	4
AP-2 complex subunit mu	Ap2m1	49965	P84091	4

150 kDa band

Protein	Gene	Molecular weight (kDa)	UniProt accession number	Number of identified peptides
Peripherin	Prph	54349	P15331	29
Vimentin	Vim	53712	P20152	32
Desmin	Des	53522	P31001	27
Heat shock cognate 71 kDa protein	Hspa8	71055	P63017	25
Actin, cytoplasmic 1	Actb	42052	P60710	17
ATP synthase subunit alpha, mitochondrial*	Atp5f1a	59830	Q03265	16
Stress-70 protein, mitochondrial	Hspa9	73701	P38647	20
Endoplasmic reticulum chaperone BiP	Hspa5	72492	P20029	20
MICOS complex subunit Mic60	Immt	84247	Q8CAQ8	20
Actin, aortic smooth muscle	Acta2	42381	P62737	14
IgE-binding protein	Iap	63221	P03975	14
60 kDa heat shock protein, mitochondrial	Hspd1	61088	P63038	13
ATP synthase subunit beta, mitochondrial*	Atp5f1b	56265	P56480	13
NADH-ubiquinone oxidoreductase 75 kDa subunit, mitochondrial	Ndufs1	80752	Q91VD9	12
ADP/ATP translocase 1*	Slc25a4	33111	P48962	9
Heterogeneous nuclear ribonucleoprotein	Hnrnpm	77940	Q9D0E1	13
Dolichyl-diphosphooligosaccharide--protein glycosyltransferase subunit 1	Rpn1	68657	Q91YQ5	13
Lamin-B1	Lmnb1	66973	P14733	13

ADP/ATP translocase 2*	Slc25a5	33138	P51881	10
Heat shock protein HSP 90-beta	Hsp90ab1	83571	P11499	9
Succinate dehydrogenase [ubiquinone] flavoprotein subunit, mitochondrial	Sdha	73623	Q8K2B3	15
Voltage-dependent anion-selective channel protein 2*	Vdac2	32340	Q60930	8
Splicing factor, proline- and glutamine-rich	Sfpq	75508	Q8VIJ6	9
Glyceraldehyde-3-phosphate dehydrogenase	Gapdh	36072	P16858	8
Elongation factor 1-alpha 1	Eef1a1	50424	P10126	8
Trifunctional enzyme subunit alpha, mitochondrial	Hadha	83302	Q8BMS1	11
Junction plakoglobin	Jup	82490	Q02257	12
Probable ATP-dependent RNA helicase DDX17	Ddx17	72981	Q501J6	10
ATP-dependent RNA helicase DDX3X	Ddx3x	73455	Q62167	15
Alpha-enolase	Eno1	47453	P17182	9
Protein disulfide-isomerase A3	Pdia3	57099	P27773	10
Voltage-dependent anion-selective channel protein 1	Vdac1	32502	Q60932	4
Probable ATP-dependent RNA helicase DDX5	Ddx5	69760	Q61656	11
Cadherin-13	Cdh13	78536	Q9WTR5	5
4F2 cell-surface antigen heavy chain	Slc3a2	58414	P10852	9
Golgi membrane protein 1	Golm1	44470	Q91XA2	6
Elongation factor 1-alpha 2	Eef1a2	50764	P62631	6
Dihydrolipoyllysine-residue acetyltransferase component of pyruvate dehydrogenase complex, mitochondrial	Dlat	68469	Q8BMF4	7
Nicastrin	Ncstn	79127	P57716	4
Nucleolin	Ncl	76734	P09405	5
Caveolae-associated protein 2	Cavin2	46792	Q63918	4
Heat shock protein HSP 90-alpha	Hsp90aa1	85134	P07901	6
Voltage-dependent anion-selective channel protein 3	Vdac3	31076	Q60931	4
Electrogenic aspartate/glutamate antiporter SLC25A12, mitochondrial	Slc25a12	74922	Q8BH59	5
Cell surface glycoprotein MUC18	Mcam	72470	Q8R2Y2	6
Pyruvate kinase PKM	Pkm	58378	P52480	4
Phosphate carrier protein, mitochondrial	Slc25a3	40063	Q8VEM8	6
T-complex protein 1 subunit beta	Cct2	57783	P80314	4
Monocarboxylate transporter 1	Slc16a1	53860	P53986	4
Neutral amino acid transporter	Slc1a5	59243	P51912	5

B(0)				
Cytochrome b-c1 complex subunit 1, mitochondrial	Uqcrc1	53446	Q9CZ13	7
Calnexin	Canx	67635	P35564	5
Ras GTPase-activating protein-binding protein 1	G3bp1	51854	P97855	4
Heterogeneous nuclear ribonucleoprotein L	Hnrnpl	64550	Q8R081	6
Non-POU domain-containing octamer-binding protein	Nono	54620	Q99K48	6
Heparan-alpha-glucosaminide N-acetyltransferase	Hgsnat	73313	Q3UDW8	4
Spectrin beta chain, erythrocytic	Sptb	245897	P15508	5
60S ribosomal protein L4	Rpl4	47409	Q9D8E6	6
Cytochrome c1, heme protein, mitochondrial	Cyc1	35533	Q9D0M3	4
T-complex protein 1 subunit delta	Cct4	58543	P80315	6
Heterogeneous nuclear ribonucleoproteins A2/B1	Hnrnpa2b1	37437	O88569	5
Nexilin	Nexn	72349	Q7TPW1	5
Glucose-6-phosphate isomerase	Gpi	62955	P06745	4
Nuclear autoantigenic sperm protein	Nasp	84188	Q99MD9	5
L-lactate dehydrogenase A chain	Ldha	36817	P06151	5
Vascular cell adhesion protein 1	Vcam1	82406	P29533	5
Sodium/potassium-transporting ATPase subunit beta-1	Atp1b1	35571	P14094	5
Solute carrier family 2, facilitated glucose transporter member 1	Slc2a1	54292	P17809	4
Far upstream element-binding protein 2	Khsrp	77184	Q3U0V1	4
Isocitrate dehydrogenase [NADP], mitochondrial	Idh2	51330	P54071	4
Heterogeneous nuclear ribonucleoprotein U-like protein 2	Hnrnpul2	85515	Q00PI9	4

1. Gizak, A.; Pirog, M.; Rakus, D. Muscle FBPase Binds to Cardiomyocyte Mitochondria under Glycogen Synthase Kinase-3 Inhibition or Elevation of Cellular Ca 2+ Level. *FEBS Letters* **2012**, *586*, 13–19, doi:10.1016/j.febslet.2011.11.032.
