# Supplementary material for: Disruption of Fructose 1,6-Bisphosphatase 2 Proximity to MIC60 Correlates with Mitochondrial Ultrastructural Changes
Source: Cells. 2026 May 20;15(10):942. doi: 10.3390/cells15100942 (PMC13204092; doi:10.3390/cells15100942)
Supplement: Supplementary file 1 [file cells-15-00942-s001.zip › Supplementary Material 1.pdf]

Supplementary Materials 1

a) Delivery of dimeric FBP2 mutant protein into HL-1 cardiomyocytes - original image of the WB membrane and quantification of the signal.

Protein delivery success was determined by Western-blot analysis using mouse anti-FBP2 antibodies (BioDavids, Regensburg, Germany) and rabbit anti-β-actin antibody (cat. no. A2066; Merck, Darmstadt, Germany) as a loading control. Appropriate secondary anti-bodies were used: goat anti-rabbit IgG (cat. no. A9044; Merck, Darmstadt, Germany) and goat anti-mouse IgG (cat. no. A9309; Merck, Darmstadt, Germany). Quantitative analysis (using ImageJ/FiJi Plot Lanes function) revealed an approximately 2-fold increase in total FBP2 signal after transfection relative to control cells.

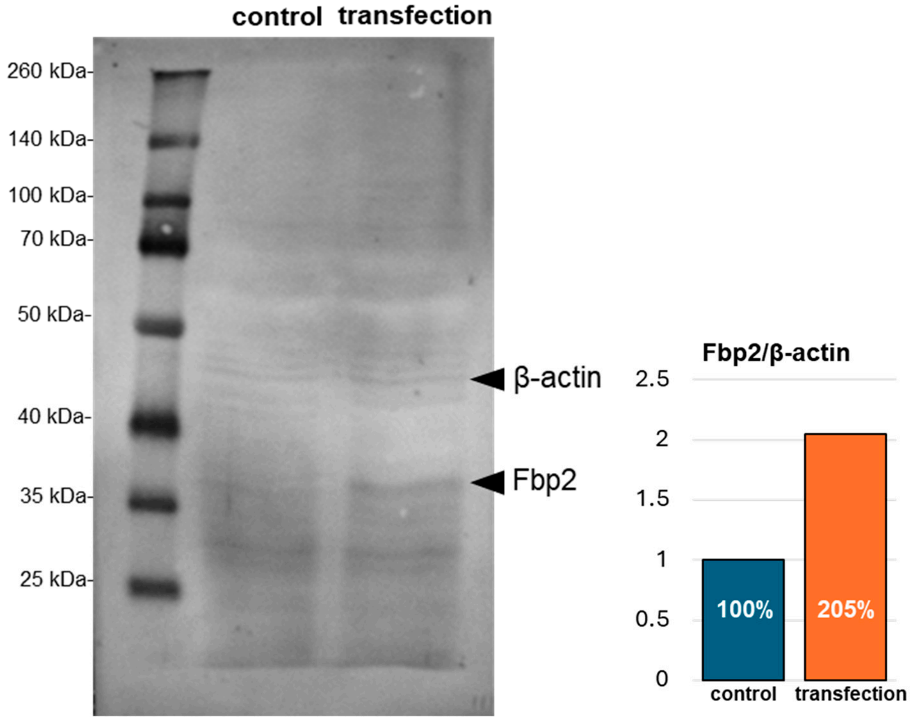

b) Original images of WB membranes and silver-stained gel (presented in Figure 3 of the main text).

For Western blotting, three lanes were used: two identical protein samples (positioned on the left and right in the image), separated by a molecular weight marker lane (middle lane). The gel was subsequently cut into two parts at the level of the molecular weight markers, and proteins from the resulting gel sections were independently transferred onto Western blot membranes. This approach was intentionally chosen to facilitate accurate alignment and identification of bands at identical molecular weights on both WB membranes. The membranes were subsequently probed with two different antibodies: against beta-tubulin (left membrane) and against FBP2 (right membrane).

The aim of this experiment was to identify proteins interacting with the FBP2–tubulin complex; therefore, all protein complexes with molecular weights equal to (~90 kDa) or lower than that of this complex were not relevant for the analysis. Accordingly, the gel region below 70 kDa was removed prior to Western blot procedure to minimize the volume of antibodies required during immunodetection.

Protein sample Markers Protein sample

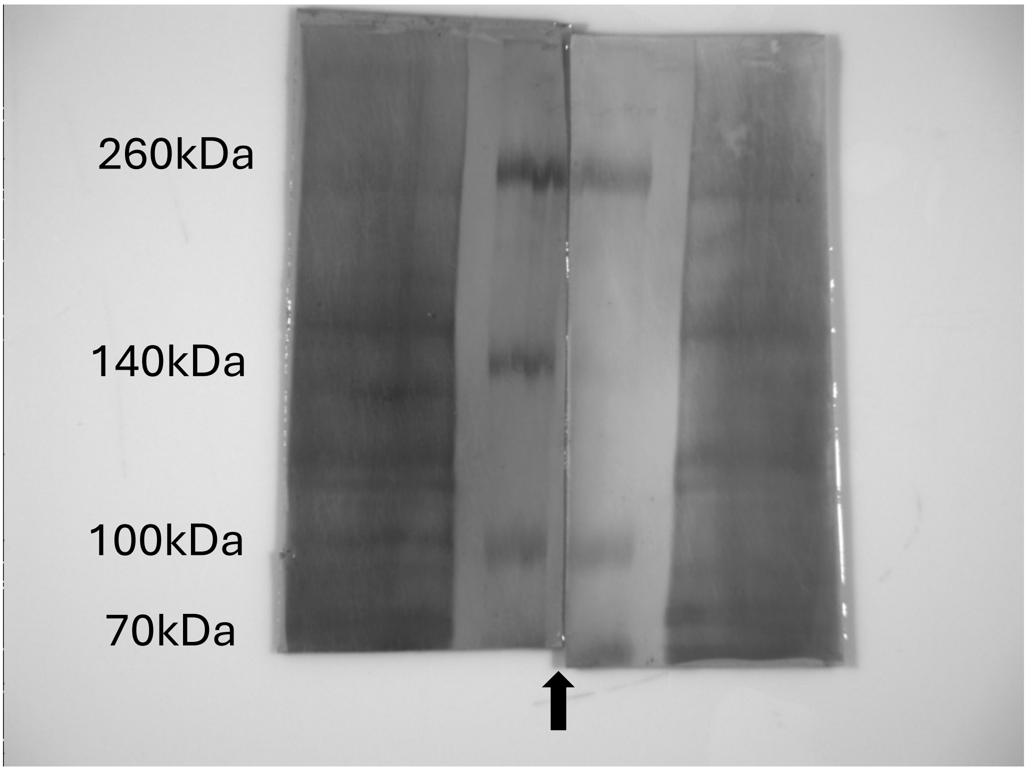

Two Western blot membranes placed side by side. The junction between the membranes is indicated by an arrow.

For electrophoresis and silver staining, only two lanes were used, corresponding to those visible in the image. No protein samples were loaded into the remaining lanes; therefore, these empty lanes were excised prior to staining, as was the gel region below the 70 kDa marker (for the reasons described above).

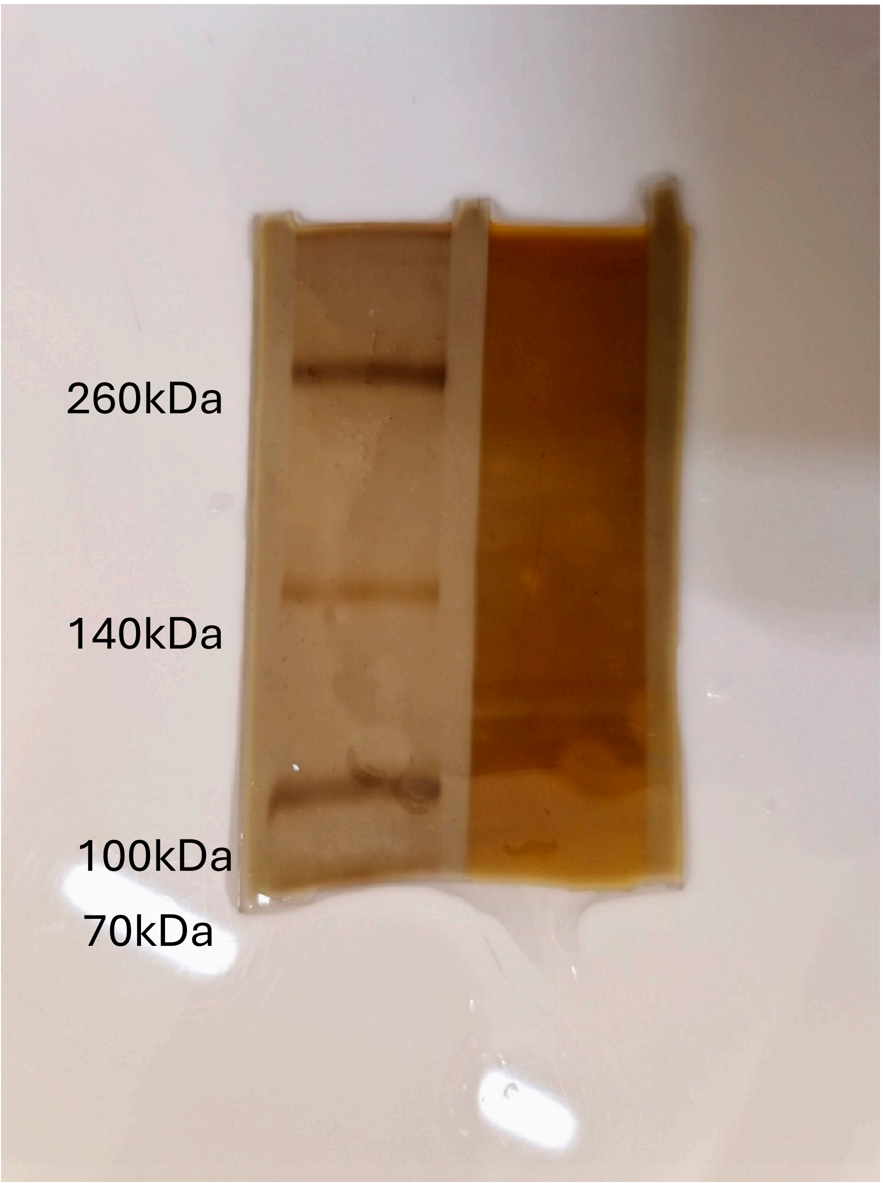

Silver-stained gel
